# Prévalence de la consommation de la chicha chez les élèves et étudiants du district sanitaire de Labé (Guinée) en 2022

**DOI:** 10.11604/pamj.2026.53.63.50481

**Published:** 2026-02-06

**Authors:** Boniface Kpamou, Bakary Oulare, Ansoumane Oulare, David Cherif, Alioune Camara

**Affiliations:** 1 Direction Régionale de la Santé de Labé, Ministère de la Santé et de l'Hygiène Publique, Labé, Guinée,; 2 Direction Préfectorale de la Santé de Lélouma, Ministère de la Santé et de l'Hygiène Publique, Lélouma, Guinée,; 3 Service d'Hématologie, Centre Hospitalier Universitaire Ignace Deen, Conakry, Guinée,; 4 Programme National de Lutte contre le Paludisme, Conakry, Guinée.

**Keywords:** Fréquence, facteurs associés, consommation, chicha, Labé, 2022, Frequency, associated factors, consumption, hookah, Labé, 2022

## Abstract

**Introduction:**

cette étude vise à évaluer la prévalence de la consommation de chicha et ses facteurs associés parmi les élèves et étudiants de la préfecture de Labé.

**Méthodes:**

une étude transversale a été conduite du 28 mars au 30 août 2022. Les données ont été collectées via un questionnaire structuré administré à 1043 participants dans 4 établissements d'enseignement secondaire et 2 établissements d'enseignement supérieur de la ville de Labé. Les données collectées ont été saisies dans Excel avant d'être analysées par l'aide du logiciel SPSS version 26.

**Résultats:**

un total de 1043 élèves et étudiants a participé à cette étude. Environ 22,7% des participants dont 55,7% chez les élèves et 44,3% chez les étudiants ont déclaré avoir consommé de la chicha. La prévalence de consommation régulière était de 2,7%. En outre, l'étude a révélé une plus grande prévalence parmi les hommes que parmi les femmes, avec des distinctions socioculturelles toujours marquées dans les habitudes tabagiques. L'analyse des différences dans la distribution du phénomène étudié a montré une consommation plus fréquente chez les hommes, les personnes âgées de plus de 18 ans et celles vivant en milieu urbain. Le fait d'appartenir à un groupe d'amis consommateurs était également significativement lié à la consommation de chicha, tandis que la scolarisation en établissement public ou privé n'avait pas d'influence.

**Conclusion:**

cette étude met en avant les dangers du tabagisme par chicha, qui devient une préoccupation croissante pour les jeunes générations.

## Abstract (en)

**Introduction:**

this study aims to assess the prevalence of hookah use and its associated factors among pupils and students in the prefecture of Labé.

**Methods:**

a cross-sectional study was conducted from March 28 to April 30, 2022. Data were collected using a structured questionnaire administered to 1,043 participants in four secondary schools and two higher education institutions in the city of Labé. The data collected were entered into Excel before being analysed using SPSS version 26 software.

**Results:**

a total of 1,043 pupils and students participated in this study. Approximately 22.7% of participants, including 55.7% of pupils and 44.3% of students, reported having used shisha. The prevalence of regular use was 2.7%. In addition, the study revealed a higher prevalence among men than among women, with socio-cultural distinctions still evident in smoking habits. Analysis of differences in the distribution of the phenomenon studied showed more frequent consumption among men, people over the age of 18, and those living in urban areas. Belonging to a group of friends who were consumers was also significantly linked to hookah consumption, while attending public or private school had no influence. **Conclusion:** this study highlights the dangers of hookah smoking, which has become an increasing concern for younger generations.

## Introduction

Dans le monde, le tabagisme par le narguilé ou la chicha est une cause majeure de décès prématurés et évitables. Plus de huit millions de personnes meurent des suites de maladies liées à la consommation de tabac, ce qui représente un coût de 1,4 milliard de dollars pour l'économie mondiale [1]. La chicha, méthode traditionnelle de consommation de tabac, a gagné en popularité auprès des jeunes et des étudiants, et prend actuellement des proportions alarmantes [2]. Les risques liés à la chicha sont souvent minimisés en raison de diverses idées reçues, comme l'utilisation d'arômes dans les produits du tabac et la fausse croyance selon laquelle la chicha serait saine car les substances nocives du tabac seraient filtrées par l'eau du réservoir [3]. Alors que, la chicha, tout comme la cigarette, est susceptible de provoquer des maladies telles que le cancer, des troubles respiratoires et gastro-intestinaux, des maladies pulmonaires chroniques et des maladies cardiovasculaires [4,5]. Une consommation régulière de chicha peut entraîner également une dépendance à la nicotine [6,7]. Il constitue également un grave problème de santé publique mondiale, en ce sens que c'est un produit toxique qui tue quasiment la moitié de ses consommateurs et un grand nombre de non-fumeurs exposés à la fumée. Son expansion semble exponentielle et se renouvelle à chaque génération [8]. À l'échelle mondiale, le tabac tue chaque année plus de 7 millions de personnes, et ce chiffre pourrait atteindre 8 millions d'ici 2030 [8]. Parmi les utilisateurs du narguilé, cette pratique connaît une popularité croissante, notamment chez les jeunes, alimentant des préoccupations majeures de santé publique.

Cependant, il est alarmant de constater que, malgré l'ignorance des jeunes quant à ces risques nocifs, très peu d'interventions de santé publique les ont ciblés. Aux États-Unis, la *Food and Drug Administration* (FDA) n'impose pas de réglementation stricte concernant la production, la distribution, la commercialisation et la vente de narguilés [9]. En Afrique, l'expansion de la consommation de chicha est notable, en particulier dans les milieux scolaires et universitaires. Par exemple, la prévalence tabagique en milieu secondaire au Maroc varie entre 8,8% et 15,4% [10]. En Guinée, une enquête dans les lycées privés de la commune de Dixinn a révélé que 50,8% des élèves consommaient de la chicha, un chiffre alarmant illustrant l'ampleur du problème.

À Labé, bien que la consommation de chicha soit en hausse, aucune étude n'a encore été menée pour évaluer ce phénomène en milieu scolaire. Ce manque de données entrave la mise en place de politiques de santé scolaire et universitaire adaptées pour lutter contre cette pratique et ses conséquences sur la santé des adolescents et jeunes. L'objectif de cette étude était d'évaluer la prévalence de la consommation de chicha et ses facteurs associés parmi les élèves, lycéens et étudiants de la préfecture de Labé.

## Méthodes

**Figure 1:**
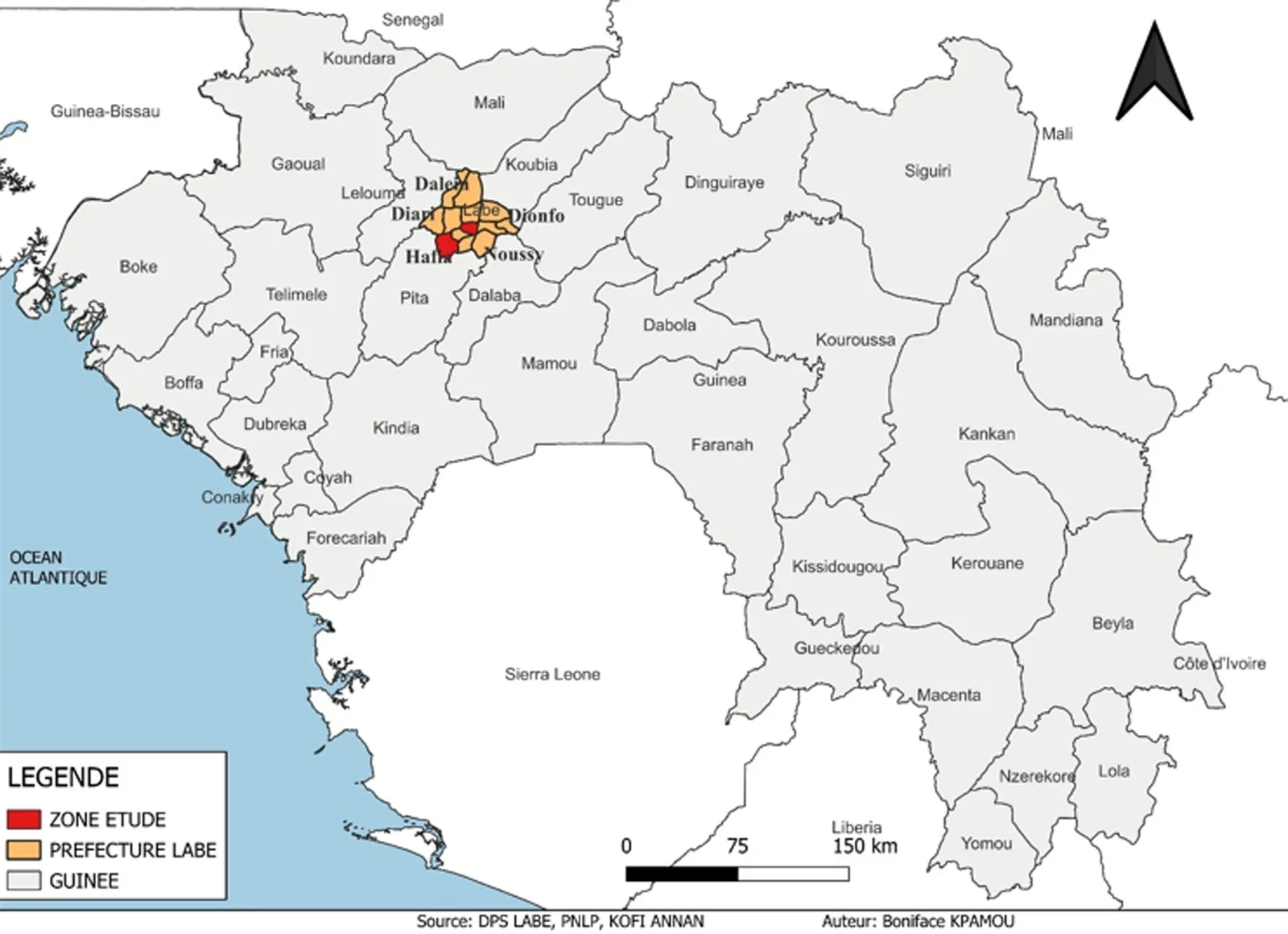
zone d'étude de la consommation de chicha, préfecture Labé, 2022

**Cadre de l'étude:** cette étude a été réalisée dans les structures scolaires de la préfecture de Labé, située dans la région administrative du même nom en Guinée. Labé couvre une superficie de 3 991 km^2^ pour une population estimée à 401 215 habitants en 2022. Elle compte 18 établissements d'enseignement secondaire et supérieur, répartis entre lycées, collèges et universités. Parmi ceux-ci, six établissements (quatre lycées et collèges, ainsi que deux universités) ont été sélectionnés de manière aléatoire pour assurer une représentativité des différents niveaux d'enseignement (Figure 1).

**Conception de l'étude:** il s'agissait d'une étude transversale descriptive réalisée du 28 mars au 30 août 2022. La population cible comprenait les élèves et étudiants inscrits dans les écoles et universités (lycée Wagon Bouro, collège Thyndel, collège Saint André, lycée Yassine Diallo, université Hafia et université Ahmadou Dieng). Les élèves et étudiants inscrits dans l'un des établissements d'enseignement public ou privé ayant consenti de façon libre et éclairée ont participé à cette étude.

### Variables étudiées

Cette recherche incluait: a) variable dépendante: consommation de chicha (Oui/Non); b) variables explicatives: caractéristiques sociodémographiques: âge, sexe, lieu de résidence (urbain/rural), type d'établissement fréquenté (collège, lycée, université, public/privé).

**Facteurs associés:** a) consommation de tabac et autres substances; b) fréquentation de pairs consommateurs de chicha ou de tabac; c) accès et facilité d'achat de la chicha.

**Collecte des données:** les données ont été recueillies à l'aide d'un questionnaire auto-administré, distribué en format papier par les enquêteurs. Le questionnaire a été élaboré sur la base d'une revue de la littérature et adapté au contexte de l'étude. Afin d'assurer sa validité et sa fiabilité, le questionnaire a été prétesté sur un échantillon de 30 étudiants ne faisant pas partie de l'étude principale. Ce pré-test a permis d'évaluer la clarté des questions, la compréhension des répondants et la durée moyenne de réponse (estimée entre 15 et 20 minutes). Les ajustements nécessaires ont été effectués avant son administration définitive.

**Analyse des données:** les données ont été saisies et nettoyées dans Excel, puis analysées avec SPSS version 26. Les variables quantitatives ont été décrites par des médianes et des étendues. Les variables qualitatives ont été exprimées en proportions. Les associations statistiques ont été testées à l'aide du test du chi carré (ou test exact de Fisher) pour une valeur de p < 0,05 considérée comme significative.

**Considérations éthiques:** l'étude a été approuvée par le comité national d'éthique pour la recherche en santé, et l'accord des autorités éducatives et sanitaires a été obtenu. Le consentement éclairé des participants a été recueilli, et toutes les données ont été traitées anonymement.

## Résultats

**Caractéristiques générales de la population étudiée:** au total 1043 questionnaires ont été recueillis dans 6 établissements d'enseignement publics et privés dans la ville de Labé sur 1044 prévus soit 99,9%: 347 étudiants et 696 avec 53,6% d'hommes et 46,4% de femmes. Le sexe ratio de 1,15 en faveur des hommes reflète une tendance fréquente dans ce type de pratiques, avec une majorité des consommateurs masculins (71%) (Tableau 1).

**Tableau 1:** réponses spécifiques des collégiens, lycéens et étudiants de Labé aux questionnaires se rapportant aux données socio-démographiques du 28 mars 2022 au 30 août 2022

Données socio-démographiques	Total	Secondaire		Université		P value
		Effectif	%	Effectif	%	
Sexe						
Féminin	46,4	356	34,1	128	12,3	**<0,001**
Masculin	53,6	340	32,6	219	21,0	
**Tranche d’âge**						
< à 18 ans	53,8	542	52,0	19	1,8	**1,45 × 10^-107^**
> à 18 ans	46,2	154	14,8	328	31,4	
**Âge (moyen ± ET) 18,1±3,1 ans**						
**Âge médiane 1^ère^ consommation 20 ans**						
**Résidence**						**<0,001**
Urbain	86,8	690	66,2	210	20,1	
Rurale	13,2	1	0,1	137	13,1	
**Lieu de vie**						**<0,001**
Famille	85,5	686	66,0	206	20,0	
Entre amis	8,9	2	0,2	91	8,7	
Seul	5,6	8	0,8	50	4,8	
**Type Établissement**						**0,878**
Publique	50,2	348	33,4	17,0	176	
Privée	49,8	348	33,4	16,4	171	

**Répartition selon la consommation de la chicha:** sur les 1043 volontaires ayant participé à l'enquête, 237 volontaires avaient au moins consommé une fois la chicha, soit une prévalence de 22,7% et 77,3% pour ceux qui n'en avaient pas consommé. L'âge médian des volontaires était de 18 ans avec les extrêmes allant de 11 ans à 28 ans et l'âge médian lors de la première consommation était de 20 ans, illustrant ainsi une initiation précoce chez une partie des participants (Tableau 1).

**Répartition selon la connaissance et les risques liés à la consommation de chicha:** la quasi-totalité des participants a déjà entendu parler du chicha (98,5%), sans différence entre collège, lycée et université. Plus de trois quarts (77,3%) considèrent la chicha comme une forme de tabagisme, avec une proportion légèrement plus élevée chez les lycéens et universitaires (Tableau 2). La croyance que l'eau filtre la fumée reste la plus fréquente: 35,8% pensent qu'elle joue un rôle réel et 48,3% un rôle partiel, surtout chez les collégiens. Par ailleurs, 89,2% reconnaissent que le partage du tuyau expose à la transmission de maladies (Tableau 2). La majorité des participants estime que la chicha est nocive pour la santé (90,9%). Toutefois, les connaissances sur les organes touchés et les maladies restent incomplètes: seuls 19,6% identifient clairement les poumons, tandis que 61,4% évoquent des atteintes diverses. Le cancer du poumon est cité par 37,7%, mais une proportion notable, surtout à l'université, déclare ne pas connaître les maladies associées (Tableau 2).

**Tableau 2:** connaissances et risques liés à la consommation de chicha chez les élèves et étudiants du district sanitaire de Labé

Connaissances liées à la chicha	Total	Collège		Lycée		Université	
	%	Effectif	%	Effectif	%	Effectif	%
**Avez-vous vu ou entendu parler de chicha**							
Oui	98,5	342	98,3	343	98,6	342	98,6
Non	1,5	6	1,7	5	1,4	5	1,4
**Considérez-vous la chicha comme une forme de tabagisme ?**							
Ne sait pas	1	1	0,3	0		9	2,6
Non	17,9	74	21,3	61	17,5	52	15
Oui	81,1	273	78,4	287	82,5	286	82,4
**Pensez-vous que l’eau joue un rôle de filtre ?**							
Aucun	35,8	179	51,4	89	25,6	105	28,2
Partiel	29,3	79	22,7	122	35,1	105	30,3
Total	19,1	29	8,3	74	21,3	96	27,7
Ne sait pas	15,8	61	17,5	63	18,1	41	11,8
**Partage du tuyau les uns avec les autres expose à la transmission des maladies ?**							
Oui	89,2	333	95,7	291	83,6	306	88,2
Non	10,2	15	4,3	57	16,4	34	9,8
Ne sait pas	0,7	0		0		7	2
**La chicha est-elle nocive pour la santé ?**							
Oui	90,9	320	92	312	89,7	316	91,1
Non	9,1	28	8	36	10,3	31	8,9
**Quels sont les organes touchés ?**							
Poumons	19,6	81	23,3	56	16,1	67	19,3
Ne sait pas	12,2	11	3,2	63	18,1	53	15,3
Cœur	6,8	35	10,1	22	6,3	14	4
Autres	61,4	221	63,4	207	59,5	213	61,4
**Quelles sont les maladies provoquées par la chicha ?**							
Cancer de poumon	37,7	162	46,6	118	33,9	113	32,6
Ne sait pas	9,4	45	12,9	82	23,6	75	21,6
HTA	1,5	1	0,3	15	4,3	0	
Autres	51,4	140	40,2	133	38,2	159	45,8

**Figure 2:**
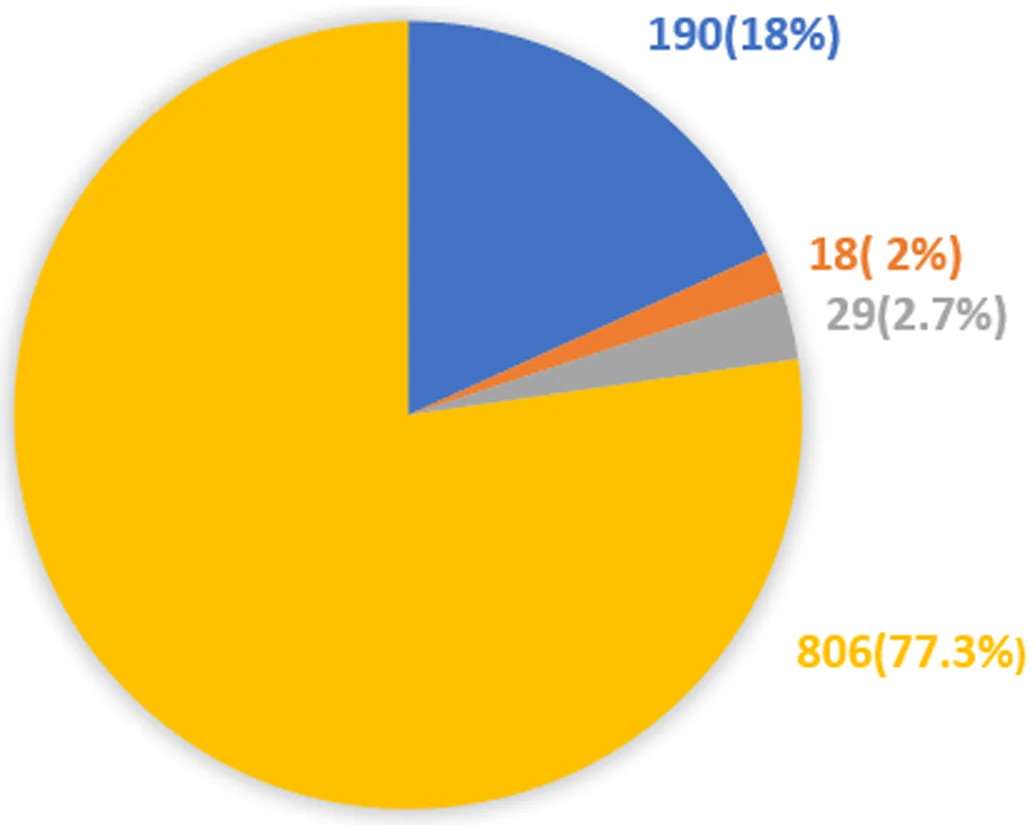
répartition des participants des établissements scolaires de Labé selon qu'ils ont consommé la chicha ou non

**Répartition des participants selon leurs habitudes de consommation de la chicha:** en ce qui concerne les habitudes de consommation, les participants privilégiaient les séances relativement courtes, généralement autour de 1 à 2 heures par session, souvent en fin de journée ou pendant le week-end (Tableau 3). Le lieu privilégié pour consommer la chicha était chez les amis, représentant près de 50% des cas, ce qui souligne une consommation socialisée et influencée par les relations interpersonnelles (Tableau 3). Dix-huit pour cent (18%) des participants des établissements scolaires de Labé disent avoir consommé occasionnellement la chicha (Figure 2).

**Tableau 3:** répartition des élèves et étudiants consommateurs de la chicha selon leurs circonstances de consommation dans le district sanitaire de Labé en 2022

Circonstances de consommation	Effectifs n=237	%
**Où consommez-vous la chicha fréquemment**		
À domicile	25	10,5
À l’extérieur du ménage	212	89,5
**Avec qui consommez-vous chicha**		
Avec des amis	224	94,5
En famille	3	1,3
Seul	10	4,2
**Moment de la journée pour fumer la chicha**		
Avant midi	5	2,1
Après-midi	232	97,9
**Moment de la semaine pour fumer la chicha**		
Quotidien	23	9,7
Week-end (festif)	214	90,3
**Charbon utilisé pour l’appareil**		
Charbon à incandescence rapide	17	7,2
Charbon classique adapté à la chicha	197	83,1
Charbon de bois pour barbecue	23	9,7
**Combinaison de la chicha à la cigarette**		
Non	219	92,4
Oui	18	7,6
**Combinaison de la chicha à l’alcool**		
Oui	24	10,1
Non	213	89,9
**Assistance à une campagne de sensibilisation sur la chicha**		
Oui	196	82,7
Non	41	17,3

**Répartition des participants selon leurs perceptions:** les perceptions liées à la chicha montraient une forte reconnaissance de sa nocivité pour la santé. Une majorité des participants considérait que la chicha affectait principalement les poumons et le cœur, tandis que 10,13% combinaient régulièrement chicha et alcool, augmentant ainsi les risques pour la santé (Tableau 3). De plus, 8% des participants associaient cette pratique à une combinaison avec la cigarette, ce qui accentue les effets négatifs sur la santé. Les campagnes de sensibilisation sur les risques de la chicha ont eu un impact significatif, avec 82,70% des participants ayant assisté à ces initiatives, bien que des défis persistent dans l'adoption de comportements plus sûrs face à cette substance (Tableau 3).

**Répartition selon les facteurs associés à la consommation de la chicha:** les résultats montraient que l'âge médian des consommateurs de chicha était significativement plus élevé que celui des non-consommateurs (20 ans contre 18 ans, p=0,0001). De plus, la majorité des consommateurs sont de sexe masculin (78,48%) comparés aux non-consommateurs (46,28%, p<0,00000) (Tableau 4). Tous les consommateurs de chicha dans notre échantillon avaient des amis, des membres de la famille ou des voisins également fumeurs. Aucun des non-consommateurs n'a rapporté une telle influence dans son entourage (p<0,0000001) (Tableau 4). Les résultats indiquaient que les jeunes vivant entre amis présentaient une prévalence plus élevée de consommation de chicha (43,01%, p=0,000000500). En revanche, ceux vivant en famille sont moins touchés (19,96%) (Tableau 4).

**Tableau 4:** comparaison des caractères selon la consommation de la chicha du 28 mars 2022 au 30 août 2022 à Labé

Caractéristiques	Consommateurs	Non consommateur	p-value
**Age médiane**	20 (13-28) ans	18 (11-28) ans	0,0001
**Sexe**			
Masculin	186 (78,48)	373 (46,28)	0,00000
Féminin	51 (21,52)	433 (53,72)	
**Établissement scolaire**			
Ahmadou Dieng	43 (18,14)	128 (15,88)	0,000000648
Hongo M'Bouro	41 (17,30)	133 (16,50)	
Saint André	28 (11,81)	146 (18,11)	
Thindel	18 (7,59)	156 (19,35)	
Université Labé	62 (26,16)	114 (14,14)	
Yacine Diallo	45 (18,99)	129 (16,00)	
**Entourage fumeur**			
Oui	237 (100)	0 (0)	< 0,0000001
Non	0 (0)	806 (100)	
**Mode de vie**			
Entre amis	40 (43,01)	53 (56,99)	0,000000500
En famille	178 (19,96)	714 (80,04)	
Seul	19 (32,76)	39 (67,24)	
**Plus nocive entre cigarette et chicha**			
Chicha	149 (24,96)	448 (75,04)	0,000002364
Cigarette	51 (32,28)	107 (67,72)	
Ne sait pas	37 (12,85)	251 (87,15)	
**Combinaison de la chicha à la cigarette**			
Fumeur	18 (66,67)	9 (33,33)	0,0000005371
Non-Fumeur	219 (21,56)	797 (78,44)	
**Consommation de la chicha combinée à l’alcool**			
Alcoolique	24 (57,14)	18 (42,86)	0,0000005150
Non Alcoolique	213 (21,28)	788 (78,72)	
**Organes susceptibles d’être atteints**			
Cœur	222 (32,55)	460 (67,44)	0,03181
Poumon	271 (32,22)	570 (67,77)	
Foie	3 (37,5)	5 (62,5)	
Estomac	78 (32,36)	163 (67,63)	
œsophage	53 (37,06)	90 (62,93)	
Pénis	2 (100)	0 (0)	
Yeux	1 (100)	0 (0)	
Vagin	1 (100)	0 (0)	
Dents	106 (41,89)	147 (58,10)	
Ne sait pas	40 (31,49)	87 (68,50)	
**Maladies susceptibles d’être provoquées**			
Cancer de poumon	245 (32,19)	516 (67,80)	0,1208
Cancer de gorge	107 (37,54)	178 (62,45)	
Cancer de l’estomac	49 (34,75)	92 (65,24)	
Cancer buccal	38 (42,22)	52 (57,77)	
HTA	24 (26,37)	67 (73,62)	
Cancer de la vessie	17 (40,47)	25 (59,52)	
Diabète	11 35,48)	20 (64,51)	
Ne sait pas	57 (28,21)	145 (71,78)	

Parmi les consommateurs, 24,96% pensaient que la chicha est plus nocive que la cigarette, contre 75,04% des non-consommateurs qui partageaient cette perception (p=0,000002364). De plus, la consommation combinée avec d'autres substances comme la cigarette (66,67%) et l'alcool (57,14%) était significativement plus fréquente chez les consommateurs (p= 0,0000005371, p= 0,0000005150) (Tableau 4). Les consommateurs associaient la chicha à plusieurs maladies, notamment le cancer du poumon (32,19%) et le cancer de la gorge (37,54%). Les organes les plus fréquemment cités comme affectés étaient les poumons (32,22%) et les dents (41,89%, p=0,03181) (Tableau 4).

## Discussion

Les résultats de notre étude sur la consommation de chicha révélaient des prévalences similaires à celles observées dans d'autres régions, bien que les contextes culturels et sociétaux diffèrent. La prévalence de 22,7% observée chez les participants de notre étude est plus élevée par rapport à certaines études internationales, telle que celle de Lasebikan *et al*. [11] au Nigeria (7,1%) et inférieure à celle de Saravanan *et al*. [12] aux Émirats arabes unis (38,9%). Ces écarts pourraient s'expliquer par l'effet de la mondialisation, la curiosité, le plaisir et l'ignorance et par des différences dans les comportements socioculturels liés au tabagisme, la disponibilité des produits et les campagnes de sensibilisation dans chaque région.

En comparant notre étude à d'autres recherches, il est évident que la consommation de chicha reste plus élevée chez les hommes que chez les femmes, une tendance observée dans de nombreuses études similaires. Le sexe masculin était le plus représenté avec 559 (53,6%), tandis que le sexe féminin n'était que de 484 (46,4%). Ces résultats sont comparables à ceux trouvés chez Wayzani *et al*. [13] publiés en 2015 au Sénégal où ils ont trouvé 848 garçons (51,3%) et 806 filles (48,7%). Nous avons constaté que les hommes sont plus représentés que les femmes, Cela pourrait s'expliquer par le fait qu'il est inacceptable et mal vu par nos mœurs que les femmes fument. Mais il est aussi important de souligner que le tabagisme à la chicha gagne du terrain du fait de sa mondialisation et de l'acquisition croissante par les filles. Quant aux lieux de consommation, nos résultats montraient une prédominance de la consommation entre amis, avec 50% des participants favorisant ce contexte. Cette tendance est similaire à ce qui a été observé dans l'étude de Ward *et al*. [14], où 35,2% fumaient habituellement à la maison ou chez un ami, et 42,1% fumaient habituellement à la fois à la maison et dans les cafés. Les perceptions de la nocivité de la chicha parmi les participants indiquaient une conscience croissante des risques associés à sa consommation. La majorité des participants à notre enquête considéraient la chicha comme nocive, avec 33,8% des collégiens et 33,3% des universitaires l'identifiant comme une forme de tabagisme. Bani *et al*. [15] ont ressorti dans leur étude qu'environ 40,2% des fumeurs estiment que ces cigarettes leur procurent du plaisir. Cependant, en 2019 à Niamey, 33,5% des étudiants ne considéraient pas la chicha comme du tabagisme, et 55,3% ignoraient qu'elle était plus nocive que la cigarette [16]. La popularité croissante de la chicha s'explique par son association avec les lieux de loisirs et les tendances modernes. Malgré une bonne connaissance générale des risques, les enquêtés ont identifié les poumons comme l'organe le plus susceptible d'être atteint (19,6%), et le cancer du poumon comme la principale maladie associée (37,7%). Ces résultats concordent avec ceux de Joachim *et al*. [17] en 2011, qui ont identifié le cancer du poumon et les maladies cardiovasculaires comme principaux effets de la consommation de tabac. Cette prédominance s'explique par le passage direct de la fumée dans les poumons, exposant cet organe en premier avant que d'autres systèmes ne soient touchés.

Notre étude révélait que 22,7% des participants consommaient la chicha, répartis entre 18% de fumeurs occasionnels, 2% de fumeurs quotidiens et 2,7% de fumeurs réguliers. Ces chiffres sont supérieurs à ceux observés au Mali en 2014 par Sangho *et al*. [18], qui étaient de 14% de consommateurs. Cette augmentation de la consommation pourrait s'expliquer par l'association de la chicha à la culture des jeunes, renforcée par l'attrait de sa fumée aromatisée. De plus, la popularité croissante des chichas dans les bars et clubs, où elles sont souvent perçues comme une alternative plus saine à la cigarette, contribue également à ce phénomène. Pour identifier les facteurs associés, nous avons utilisé le test du chi carré (test exact de Fisher ou test de Pearson), en considérant une valeur p inférieure à 5% comme statistiquement significative. Il a été constaté que le sexe masculin, un âge supérieur à 18 ans, le fait de vivre en milieu urbain, ainsi que l'appartenance à un groupe d'amis étaient des facteurs significativement associés à la consommation de chicha. En revanche, la nature de l'établissement scolaire (public ou privé) n'a pas montré d'influence significative sur cette pratique.

**Limites de l'étude:** malgré la richesse des données recueillies, plusieurs limites doivent être soulignées. Premièrement, la méthodologie d'auto-déclaration pourrait engendrer des biais de réponse, notamment des sous-estimations ou surestimations des pratiques. Deuxièmement, bien que l'échantillon soit représentatif des établissements de Labé, il n'inclut pas d'autres villes de Guinée, limitant ainsi la généralisation des résultats. Enfin, la durée et la complexité de l'enquête ont pu impacter la qualité des réponses obtenues, notamment en termes de fatigue des participants.

## Conclusion

Notre étude démontre que la consommation de chicha parmi les jeunes élèves et étudiants constitue une préoccupation majeure de santé publique avec une prévalence de 22,7%. Il est évident que cette pratique, bien qu'émergente, touche une large tranche de la population jeune, indépendamment du sexe ou de l'âge. Cette forme de tabagisme représente une menace croissante pour la santé des jeunes, d'autant plus qu'elle prend de l'ampleur dans les écoles et universités de Labé. Les résultats de cette étude mettent en évidence la nécessité urgente de sensibilisation et de mesures concrètes pour prévenir cette addiction, non seulement à Labé mais également dans d'autres préfectures de la Guinée. Des recherches supplémentaires sont essentielles pour mieux comprendre les facteurs sous-jacents et orienter les politiques de santé publique adaptées.

### Etat des connaissances sur le sujet

- La chicha expose ses utilisateurs à des risques comparables, notamment des maladies pulmonaires;
- Cardiovasculaires et des cancers, ainsi qu'à des complications graves comme la broncho-pneumopathie chronique obstructive (BPCO);
- La consommation de la chicha constitue également un problème majeur de santé publique.

### Contribution de notre étude à la connaissance

- Cette étude nous permet de connaître le motif réel de la consommation de la chicha à Labé;
- Cette étude fournit des données de prévalence sur la consommation de chicha à Labé et le profil des consommateurs.

## Remerciements

Les auteurs remercient chaleureusement les docteurs Saidouba Chérif Camara, Karifa Sitan Camara, Maladho Diaby et Haby Diallo pour leur accompagnement dans l'élaboration du manuscrit.

## References

[1] Organisation mondiale de la Santé Rapport de l'OMS sur l'épidémie mondiale de tabagisme, 2021: les produits nouveaux et émergents.

[2] Aboaziza E, Eissenberg T (2015). Waterpipe tobacco smoking: what is the evidence that it supports nicotine/tobacco dependence?. Tob Control.

[3] Babaie J, Ahmadi A, Abdollahi G, Doshmangir L (2021). Preventing and controlling water pipe smoking: a systematic review of management interventions. BMC Public Health.

[4] Travers MJ, Rivard C, Sharma E, Retzky S, Yucesoy B, Goniewicz ML (2020). Biomarkers of Exposure among USA Adult Hookah Users: Results from Wave 1 of the Population Assessment of Tobacco and Health (PATH) Study (2013-2014). Int J Environ Res Public Health.

[5] Al Ali R, Vukadinović D, Maziak W, Katmeh L, Schwarz V, Mahfoud F (2020). Cardiovascular effects of waterpipe smoking: a systematic review and meta-analysis. Rev Cardiovasc Med.

[6] Aghdam FB, Alizadeh N, Nadrian H, Augner C, Mohammadpoorasl A (2021). Effects of a multi-level intervention on hookah smoking frequency and duration among Iranian adolescents and adults: an application of socio-ecological model. BMC Public Health.

[7] Osibogun O, Odukoya OO, Odusolu YO, Osibogun A (2020). Knowledge and risk perception of e-cigarettes and hookah amongst youths in Lagos State, Nigeria: An exploratory study. Niger Postgrad Med J.

[8] Qasim H, Alarabi AB, Alzoubi KH, Karim ZA, Alshbool FZ, Khasawneh FT (2019). The effects of hookah/waterpipe smoking on general health and the cardiovascular system. Environ Health Prev Med.

[9] Fevrier B, Vidourek RA, Privitera P (2018). Policy Implications and Research Recommendations: A Review of Hookah Use Among US College Students. J Community Health.

[10] Bennani MA, Bouchareb A, Ayad A, Bourkadi D, Ouardi A, Berrabeh Y (2022). Chicha et cigarette électronique en milieu scolaire de la ville d'Oran. Revue des Maladies Respiratoires Actualités.

[11] Lasebikan VO, Ola BA, Lasebikan TO (2019). Shisha smoking in selected nightclubs in Nigeria. Pan Afr Med J.

[12] Saravanan C, Attlee A, Sulaiman N (2019). A Cross Sectional Study on Knowledge, Beliefs and Psychosocial Predictors of Shisha Smoking among University Students in Sharjah, United Arab Emirates. Asian Pac J Cancer Prev.

[13] Wayzani M, Dia Kane Y, Thiam K, Touré NO, Mbaye FBR, Ndiaye EHM (2015). Prévalence du tabagisme dans l'enseignement moyen et secondaire dans le Département de Dakar, Sénégal [Prevalence of tobacco smoking in primary and secondary schools in the Department of Dakar, Senegal]. Rev Mal Respir.

[14] Ward KD, Kumar J, Khan Z, Jiang Y (2019). Characteristics of Waterpipe Health Warning Labels in the United States. Am J Health Behav.

[15] Bani Hani A, Mansour S, Al Smady MM, Bani Hani F, Obeidat SM, Zahran EA (2025). Waterpipe tobacco smoking in healthcare students in the University of Jordan. Front Public Health.

[16] Neino MA, Salifou IA, Issoufou MG, Daouda AH, Ouédraogo AR, Mallam AM (2019). Knowledge and attitudes of the students of the Niamey Faculty of Health Sciences on tobacco.

[17] Joachim M, Jeanrenaud C, Ecabert C, Perrin E Perception des risques liés au tabagisme: enquête auprès des jeunes de 15 à 20 ans 2011.

[18] Sangho H, Keïta AS, Kodio A, Tayeb MM, Keïta HD, Cissé MO (2014). Tabagisme en milieu scolaire dans une commune de Bamako au Mali [Smoking at school in a commune of Bamako, Mali]. Mali Med.

